# Notes and News

**Published:** 1898-01-01

**Authors:** 


### NOTES AND NEWS.

(Collected and Communicated.)

Mr. T. F. Blackwell, a governor of St. Thomas's Hospital, has contributed £1,000 for the purpose of endowing a bed in perpetuity.

The hon. secretaries of the Prince of Wales's Hospital Fund for London have received from Sir Edward Lawson 755,599 shillings and 7 pence (£37,779 19s. 7d.) being the amount received to this date in answer to the appeal on behalf of the Prince's Jubilee Hospital Fund, only the cost of collection and out of pocket expenses being deducted, no charge whatever having been made on account of the *Daily Telegraph* itself or any member of its staff.

The Diamond Jubilee Bazaar held in Leicester in November for the benefit of the Leicester Infirmary has resulted in the very handsome sum of £2,600 being handed over to the treasurer of that charity. The total receipts were £3,277 and the expenditure £676. We are informed that the Mayor's Commemoration Fund for the Leicester Infirmary amounts to nearly £10,000.

His Royal Highness the Prince of Wales, K.G., after consultation with the Council of the Prince of Wales's Hospital Fund for London, has decided to distribute the funds at his disposal for the year 1897 in the following way, viz.: £22,050, representing the annual subscriptions promised, plus the amount of interest on investments already made, which sum will be divided among the hospitals where more than 100 beds are in constant occupation. The moneys received from the sale of stamps, probably about £38,000, will be divided among such institutions as come under the classification of hospitals. The above-mentioned items, amounting together to about £60,000, which His Royal Highness will distribute this year, ought considerably to diminish the deficit which has been so long complained of. The most pressing wants of the larger hospitals are thus relieved for this year, and it is only reasonable to suppose that in future years the income of the Fund will be sufficient to meet their requirements. Wards which, for want of funds, have been closed at Guy's and St. Thomas's and other hospitals will be at once reopened, and the requirements of other hospitals will be met. The Prince of Wales's Fund will thus meet requirements which neither annual subscriptions nor Hospital Saturday and Sunday Funds have hitherto been able to deal with. His Royal Highness the Prince of Wales, in originating this Fund, was deeply impressed with the urgent wants of London hospitals. There is every reason to hope that the Fund for this year will remove much anxiety from hospital managers; and it is to be hoped that now once the public see that this Fund can grapple successfully with a great public want, they will subscribe to it annually, and will also endeavour, by organised and systematic contributions, to render the future of the Fund secure.

